# An Approach for the Optimization of Thermal Conductivity and Viscosity of Hybrid (Graphene Nanoplatelets, GNPs: Cellulose Nanocrystal, CNC) Nanofluids Using Response Surface Methodology (RSM)

**DOI:** 10.3390/nano13101596

**Published:** 2023-05-10

**Authors:** Chong Tak Yaw, Siaw Paw Koh, Madderla Sandhya, Devarajan Ramasamy, Kumaran Kadirgama, Foo Benedict, Kharuddin Ali, Sieh Kiong Tiong, Ahmed N. Abdalla, Kok Hen Chong

**Affiliations:** 1 Institute of Sustainable Energy, Universiti Tenaga Nasional (The Energy University), Jalan Ikram-Uniten, Kajang 43000, Malaysia; chongty@uniten.edu.my (C.T.Y.); siehkiong@uniten.edu.my (S.K.T.); 2 College of Engineering, Universiti Malaysia Pahang, Gambang 26300, Malaysia; deva@ump.edu.my; 3 Advanced Nano Coolant-Lubricant (ANCL), College of Engineering, Universiti Malaysia Pahang, Pekan 26600, Malaysia; kumaran@ump.edu.my; 4 Faculty of Mechanical and Automotive Engineering Technology, Universiti Malaysia Pahang, Pekan 26600, Malaysia; 5 Automotive Engineering Centre, Universiti Malaysia Pahang, Pekan 26600, Malaysia; 6 Enhance Track Sdn. Bhd., No. 9, Jalan Meranti Jaya 12, Meranti Jaya Industrial Park, Puchong 47120, Malaysia; 7 Faculty of Electrical and Automation Engineering Technology, University College TATI, Teluk Kalong, Kemaman 24000, Malaysia; kharudin@uctati.edu.my; 8 Faculty of Electronic Information Engineering, Huaiyin Institute of Technology, Huai’an 223025, China; ahmed@hyit.edu.cn; 9 College of Engineering, Universiti Tenaga Nasional (The Energy University), Jalan Ikram-Uniten, Kajang 43000, Malaysia; chongkh@uniten.edu.my

**Keywords:** CNC, central composite design, coefficients, correlation, energy, glycol-based graphene nanoplatelets, heat transfer, hybrid nanofluid, response surface methodology

## Abstract

Response surface methodology (RSM) is used in this study to optimize the thermal characteristics of single graphene nanoplatelets and hybrid nanofluids utilizing the miscellaneous design model. The nanofluids comprise graphene nanoplatelets and graphene nanoplatelets/cellulose nanocrystal nanoparticles in the base fluid of ethylene glycol and water (60:40). Using response surface methodology (RSM) based on central composite design (CCD) and mini tab 20 standard statistical software, the impact of temperature, volume concentration, and type of nanofluid is used to construct an empirical mathematical formula. Analysis of variance (ANOVA) is applied to determine that the developed empirical mathematical analysis is relevant. For the purpose of developing the equations, 32 experiments are conducted for second-order polynomial to the specified outputs such as thermal conductivity and viscosity. Predicted estimates and the experimental data are found to be in reasonable arrangement. In additional words, the models could expect more than 85% of thermal conductivity and viscosity fluctuations of the nanofluid, indicating that the model is accurate. Optimal thermal conductivity and viscosity values are 0.4962 W/m-K and 2.6191 cP, respectively, from the results of the optimization plot. The critical parameters are 50 °C, 0.0254%, and the category factorial is GNP/CNC, and the relevant parameters are volume concentration, temperature, and kind of nanofluid. From the results plot, the composite is 0.8371. The validation results of the model during testing indicate the capability of predicting the optimal experimental conditions.

## 1. Introduction

In the processing of diverse products, heat transfer has a significant impact on cost, production rate, and product quality. In any industrial process, improving heat transmission can result in substantial energy reserves [1]. From an energy standpoint, it is critical to cut energy consumption by changing the manufacturing process or updating the apparatus utilized for the aforementioned purposes. Industry can use proposed trials to systematically investigate the process or product variables that affect product quality. Various analyses have been conducted with the goal of improving the thermal characteristics of nanofluids [2,3]. To improve heat transfer of the systems, addition of nanoparticles is one of the current trends for improving thermal characteristics. Maxwell was the first to improve excess heat transmission of liquids by introducing compact particles in 1873 [4]. Unfortunately, this process still has a number of flaws, including pressure loss, precipitation, corrosion, and contaminants [5]. However, as the expertise has progressed, many flaws have been identified and addressed by many scholars.

A particular class of fluid called nanofluids excels at heat transmission. Metal (inorganic) and nonmetal (organic) nanoparticles which are lesser over 100 nm are dispersed and suspended in a base fluid such as water or ethylene glycol. Enhancing heat transfer is performed in various ways. Disrupting boundary layers and enhancing thermo-physical properties, i.e., thermal conductivity with introducing solid compact particles into conventional fluid are examples of such strategies. For the first time, the fluids mentioned were referred to as nanofluids. Out of several physical characteristics of nanofluids, thermal conductivity is a crucial one to research. To calculate the thermal conductivity and viscosity of nanofluids, numerous experimental and numerical studies have been conducted. Esfe, Saedodin [6] conducted experimental investigation on the thermal conductivity of a magnesium oxide/ethylene glycol nanofluid. Using an ANN model, they suggested a typical model based on temperature, fluid concentration, and size of the particle [7]. They also provided two new viscosities of nanofluid correlations which are similar to volume concentration of fluid of the nanoparticle and temperature, and investigated the thermal conductivity of ethylene glycol-based nanofluids and viscosity, which also comprises nanoparticles of magnesium (OH)^2^ [8]. Experimentally, Esfe, Saedodin [7] investigated nanofluids thermal conductivity containing nanoparticles which are of Al_2_O_3_ at an average 5 nm diameter and suspended in a liquid. Al_2_O_3_ and water composition thermal conductivity is tested at temperatures ranging from 26 to 55 °C. The findings revealed that increasing temperature of nanofluids increased their thermal conductivity significantly at particular volume concentration. Putra, Roetzel [9] investigated the thermal conductivity of an Al_2_O_3_ and water nanofluid with nanoparticles averaging 131 nm in size. Thermal conductivity is measured via an approach identified as steady-state parallel plates. The outcomes indicated that reaching a concentration of 4% of nanofluid boosted the nanofluid’s thermal conductivity. Ahmadi, Ettefaghi [10] investigated the thermal conductivity of MWCNT-oil by using narrow compact solid variety of concentrations with a temperature of 20 °C. The thermal conductivity of the nanofluid rises with increase of solid concentration, according to their findings. The nanofluid thermal conductivity improved by 20%, based on the experts. Asadi and Asadi [11] studied the rheological behavior of a WCNT/MgOSAE50 hybrid nano-lubricant in another published study. Researchers examined the influence of temperature along with solid volume fraction which affected the nano-dynamic lubricant viscosity, identified that regardless of temperature, increasing the solid volume fraction increased the dynamic viscosity. By altering nanoparticle volume fraction and temperature range by 25–50 °C and 0–3.45%, Toghraie, Chaharsoghi [12] investigated the influence of temperature and particle concentration on the thermal conductivity of hybrid nanofluids of ZnO–TiO_2_/EG. They discovered that boosting both factors enhanced the thermal conductivity of the created hybrid nanofluid (volume fraction of particles and temperature). The article investigates the thermal conductivity of polyamide-6,6/carbon nanotube composites and the effects of polymer linkage between tubes. The results suggest that the thermal conductivity of composites can be optimized by controlling the CNT diameter and polymer linkage between tubes. The findings have potential applications in the development of high-performance thermal management materials for electronic devices and other heat transfer applications [13]. The influence of higher filler concentrations on the thermal conductivity and viscosity of nanofluids study focus on a specific range of concentrations, the behavior of nanofluids can vary at higher concentrations due to the bridging effect of polymer chains. Similarly, viscosity at high filler concentrations increases dramatically over that of bulk value and these develop the high-performance devices [14]. An article [15] provided a comprehensive review of various models used to predict the thermal conductivity of nanofluids. The article discusses each model, as well as their accuracy and applicability to different types of nanofluids. The articles [16,17,18] provided recommendations for future research.

The article by authors Murshed and De Castro [19] provides an overview of the thermal properties of nanofluids containing carbon nanotubes. The authors discuss the potential benefits of using these nanofluids in various applications, including heat transfer and energy storage. They also review the current state of research in this area, highlighting the challenges and opportunities for future development. Overall, the other articles also suggest that nanofluids have significant potential for improving thermal performance in a range of applications [20,21].

Thermal conductivity, viscosity, and specific heat capacity are notable, with thermal conductivity viscosity being most essential [22]. The majority of this research has been found to be on thermal conductivity, with just around 5% of literature works including specific heat capacity. However, experimental values for most ionic liquids are still unavailable, and data from literature frequently have unreasonably high levels of uncertainties, through differences between reported values of up to 18% [23,24]. RSM is a combination of mathematical as well as statistical methodologies that is used for improving processes whose outcomes were influenced by a variety of variables [25]. A standard meaning of response surface is provided using a graphical mathematical method. The number of tests is reduced when this statistical design is used [26]. Furthermore, all quadratic regression model coefficients along with collaboration issues become estimable. The response surface model (RSM) was used to categorize association of thermal conductivity as well as viscosity, on directive to explore impact of the key components (temperature, volume concentration, and nanofluid type) and collaboration influences.

## 2. Methodology

In this study, the methods, supplies, and tools used to analyze the water- and ethylene glycol-based graphene nanoplatelets (GNPs) and hybrid nanoparticle nanofluids are described.

### 2.1. Nanofluid Preparation and Evaluation of Thermal Characteristics

The graphene nanoplatelets (GNPs) employed in this investigation were of 800 m^2^/g specific surface area (SSA) and were purchased from Nanografi nanotechnology-Turkey using the following requirements: purity: 99.9%, size: 3 mm, and diameter: 1.5 m. MY Biomass Sdn. Bhd. Malaysia purchased crystalline nanocellulose at the same time. Because of its hydrophilic nature, CNC proved difficult to extract in powder form from the generated pulp. A spray drying approach with a tiny fan was employed in dry powder form for CNC processing. When the pulp or suspensions are in contact by hot air from the spray dryer’s nozzle opening, the moisture quickly evaporated, resulting in stable CNC flake. CNC flakes were collected and ground into powder. In the Advanced Automotive Liquid Lab of the Faculty of Mechanical Engineering, University Malaysia Pahang, the needed graphene nanoplatelets and CNC nanofluid were successfully prepared. GNPs with varying volume concentrations of 0.01 percent, 0.05 percent, 0.1 percent, and 0.2 percent were weighed using an Internal Sartorius Analytical Balance (Model: BSA224S-CW), then subjected to magnetic stirring and scattering for around 2–3 h in a 60:40 ethylene glycol and distilled water mixture. A 13 mm ultrasonication probe with a power outlet of 910 W and a power supply frequency range of 20 KHz was utilized (Ultrasonic Homogenizer CE ISO Processor Cell Disruptor Mixer Sonicator 25–1050 mL). Equation (1) is used to confirm the weights of nanoparticles [27].

(1) $$W_{GNP}=\left(\frac{\phi }{100-\phi }\right)*\left(\frac{\rho_{GNP/CNC}}{\rho_{bf}}\right)W_{bf}$$

(2) $$\rho_{GNP/CNC}=\frac{\phi_{GNP}\rho_{GNP}+\phi_{CNC}\rho_{CNC}}{\phi_{total}}$$

where ‘ϕ’ represents the volume concentration (%) of nanofluids and ‘W’ represents weight, ‘$\rho_{bf}$’ represents density and ‘bf’ stands for base fluid. Subscript values of GNP and CNC represent nano particles.

Due to the hydrophobic nature, and without a surfactant addition, carbon-based nanoparticles cannot disperse in base fluid [28]. It has been revealed that GNPs may be dispersed in a media using a stirrer and probe sonication without the need of surfactants [29]. To effectively disseminate and stabilize the nanoparticles, ultrasonication was used for 5 h. Similarly, preparation of hybrid nanofluid including GNPs and CNCs in a 50:50 ratio is disseminated via stirrer in a base fluid of ethylene glycol-distilled water (60:40) around 2–3 h, followed by a 5 h ultrasonication process by 50% power output. Throughout the sonication procedure, a 5 min intermission was taken after every 15 min to minimize nanofluid overheating due to particle characteristics. For hybrid nanoparticles, the density of nanoparticles was confirmed using Equation (2).

### 2.2. Thermal Conductivity and Viscosity Measurements

Various strategies for evaluating the nanofluids thermal conductivity have been tried in recent years. Transient hot wire is the most accurate and quickest of all these approaches (THW). In this research, a hot wire-type KD2-Pro (Decagon devices Inc., Pullman, WA, USA) is utilized to evaluate the thermal conductivity of GNPs/base fluid, CNC/base fluid, and GNP-/CNC-based hybrid nanofluid [30,31,32]. During the thermal conductivity evaluation, a temperature bath (WNB7-Manufactured by Memmert, Schwabcah, Germany) is used for ensuring and maintain the temperature. To reduce experimental mistakes, probe vibration must be controlled. To orient the KS-1 probe vertically in the center of sample vial, a horizontal support remained mounted adjacent to the temperature bath. To examine the reproducibility of the data, with a 5 min interval, the measurements were carried out three times in each of the intended volume fractions and temperatures.

A rheometer (Brookfield DV-I prime viscometer) is utilized to measure the viscosity value of all nanofluids with a temperature range between 20 and 50 °C by changing volumetric concentration. A circulating water jacket is attached to an RST coaxial cylinder rheometer for studying the temperature range of different applications. The Rheometer is capable of measuring viscosities ranging from 0.0001 to 5.4 × 10^6^ Pa·s and temperatures ranging from −200 to +180 °C. The experiment took place in a steady-state condition. The method of measurement was rotational measurement with a controlled shear rate. The viscosity of the base fluid was tested in order to validate the rheometer, and findings were compared to ASHREE standard data. With 15.7 mL of fluid, the viscosity is measured, and the findings are compiled in a computer connected to an RST rheometer. Data were collected twenty times, then averaged, to reduce experimental error. The Brookfield rheometer was previously used by various researchers to determine viscosity [33,34,35].

### 2.3. Uncertainity Analysis

The viscometer has an accuracy of ±3%, while the thermal analyzer device has an accuracy of ±5%. To determine the uncertainty of the measured data, the following equation is used.

Using Equation (3), where U represents the standard uncertainty, N is the number of measurements, and S is the standard deviation, the uncertainty of the dynamic viscosity of the base fluid at a temperature of 30 °C and a shear rate of 9981 s^−1^ was determined to be 3.22%. Additionally, the uncertainty of the thermal conductivity of base fluid at temperature of 30 °C was calculated to be 5.13%.

(3) $$s=\sqrt{\frac{1}{N-1}\sum_{i=1}^{N}{\left(X_{i}-X\right)}^{2}}$$

### 2.4. Response Surface Methodology

The response surface technique is a collection of rigorous mathematical and statistical operations utilized to build and optimize methods (RSM) [36]. The main use of RSM is described in unique conditions when many input variables affect certain execution phases and process quality elements. The response scale is referred to as the execution scale, while input variables are often referred to as independent variables, which engineers typically control. In order to construct a good typical model to estimate response and independent variables, this work focused on the RSM statistical modeling status. The approximation model is a tentative model that is developed built on either process or system information. The quantitative technique, including multiple regressions, is indeed a combination of very essential tools for constructing the required uncertain models for the RSM [37].

There are a number of possibilities for assessing the precision of a linear regression model, including aspects of Least Squares Estimation methods. The least possible squares approach generates an impartial estimation for the β parameter in a model of multiple linear regressions. A crucial parameter is the sum of squares of residuals, which is given as:

The variance of considerations ${(y}_{i})$ and fitted values ${\hat{y}}_{i}$, which were indicated through yields n × 1 vector of residuals [38]. The inaccuracy or residual sum of the squares is the name given to Equation (5). To calculate the total squares of the sum, Equation (6) is presented. As a result, factors of multiple determinations (R^2^) are calculated as follows Equation (7):

(4) $${SS}_{E}=\sum_{i-1}^{n}(y_{i}-{\hat{y}}_{i}{)}^{2}=\sum_{i=1}^{n}e_{i}^{2}=e^{\tau }e,\;\mathrm{where}\;e_{i}=y_{i}-{\hat{y}}_{2}$$

Since $X^{\tau }Xb=X^{\tau }y$, the calculation formula SS_E_ may manifest as:

(5) $${SS}_{E}=y^{\tau }y-b^{\tau }X^{\tau }y$$

(6) SSE=yτy−(∑yi)2i=1nn=∑i=1nyi2−(∑yi)2i=1nn

(7) $$R^{2}=1-\frac{{ss}_{E}}{{SS}_{\tau }}$$

Then, to the best of its ability, a cubic regression examines reaction variables obtained after experimentation to specify the mathematical patterns. The regression models’ competency and reliability were further investigated using analysis of variance (ANOVA). The outcomes of experiment statistics on thermal conductivity improvement along with increase of viscosity in relation to solid volume concentration along with temperature were utilized to approximate the appropriate correlation to the response surface technique. Experimental statistical data were divided into ranges of 0.01 to 0.2% and 20 °C to 50 °C for the solid volume percent and temperature, respectively.

### 2.5. Design of Experiment

Minitab is a standard geometric software system, used for creating an experimental design which is established using varied value components, and is shown in Table 1. In the design, two continuous factors and one categorical non-value factor are considered in the experiment. The central composite design (CCD) method is used for creating models with two continuous variables and one categorical variable. There are three different stages: low value (−1) and the high value (+1). Temperature (T) and volume concentration (Ø) are continuous variables, as the kind of nanofluid is categorical data. It indicates that while the type of nanofluid will be considered during the formulation of the empirical approach, it will not be included in the empirical formula. The effect of volume concentration, nanofluid type, and temperature on thermal conductivity and viscosity is investigated for about 32 experiments overall. Table 2 summarizes the layout design along with analytical data.

## 3. Results and Interpretation

### 3.1. Anova Analysis

ANOVA analysis in RSM (response surface methodology) is a statistical technique used to analyze the effects of multiple variables on a response variable. In the context of nanofluid property optimization, ANOVA analysis can be used to determine the optimal combination of variables (such as nanoparticle concentration, temperature, and pressure) that will result in the desired properties of the nanofluid. The variables that were measured are the thermal conductivity and viscosity.

The ANOVA table summarizes the statistical significance of each variable and their interactions on the response variable. This table identifies the variables that have the most significant impact on the response variable and determines the optimal combination of variables that will result in the desired properties of the nanofluid. The response model was statistically tested using analysis of variance assessment. The variance analysis is conducted utilizing the program Minitab 18. The variance analysis uses parameters such as Probability value-‘P’, Fisher’s test (F-test), and coefficient of variance (R-square) to investigate model’s suitability along with its significance. The important findings of ANOVA analysis of thermal conductivity and viscosity are shown in Table 3 and Table 4. According to the model summary stated in Table 5 and Table 6, the maximum R-squared (R^2^) and adjusted R-squared (R^2^-adj) values were identified in models which have a strong ability to correlate data (R^2^-adj). The ratio of the model’s changes for all changes is signified with the coefficient of determination, R^2^. The fitting model’s power to reflect response fluctuations such as a measure of individual variable quantity increases as R^2^ approaches one. In general, R^2^ should be at least 80% for a standard with decent fit. The model’s determination factors for thermal conductivity and viscosity in this investigation are 98.96% and 99.05% for R-squared (R^2^) and 98.60% and 98.72% for adjusted R-squared (R^2^-adj). As a result, the standard correctly associates the investigational information including both parameters. This section provides a concise description of the experimental results, their interpretation, and the experimental conclusions.

### 3.2. The Effect of Independent Variables on Responses

In this study, the impact of volume concentration, temperature, and nanofluid type on the thermal characteristics of nanofluids, including thermal conductivity and viscosity are investigated. These factors were found to be among the most significant in determining the properties of the nanofluid. To evaluate the effectiveness of the model, *p*-value is used to assess the significance of each term. Myers, Montgomery [39] claim that if the value of ‘P’ is less than 0.05, it means that this is a significant factor in the response. Table 3 displays the factors that influence thermal conductivity, including volume concentration, temperature, and nanofluid type. The table also shows the interaction between volume concentration and temperature, as well as volume concentration and the principal cause of thermal conductivity, which is the fluid type of the nanofluid. In contradiction to thermal conductivity, the effective parameter for viscosity is volume concentration, temperature, and type of fluid, as indicated in Table 4, with a two-way interaction between Vol concentration*Temperature also being an influencing factor. Table 3 and Table 4 indicate that the Vol concentration*Type of fluid interaction factor affects thermal conductivity but not viscosity. Temperature*Fluid type does not have a significant impact on either response, so it can be excluded from the model. This means that a minor interaction between the two independent variables would not affect the effectiveness.

### 3.3. The Development of an Empirical Model

Equation (8) shows the empirical relation between GNP/CNC thermal conductivity, whereas Equation (9) shows the empirical model for viscosity for GNP/CNC.

Regression Equation in Uncoded Units for thermal conductivity:

(8) $$Thermal\;conductivity=0.3786+0.3973\phi +0.001314T-0.433\phi^{2}+0.000003T^{2}-0.00377\phi \times T$$

Regression Equation in Uncoded Units for viscosity:

(9) $$Viscosity=6.782+12.63\phi -0.0526T-2.56\phi^{2}-0.000663T^{2}-0.1727\phi \times T$$

Temperature and volume concentration have a beneficial impact on thermal conductivity of GNP/CNC nanofluid, as demonstrated in Equation (8). Additionally, the coefficient of the factor is greater than T, meaning that the modifying factor has a larger influence on thermal conductivity of hybrid graphene nanoplatelets/cellulose nanocrystal (GNP/CNC) nanofluid than factor T. Factor T has an adverse significance on viscosity of GNP/CNC nanofluid, while factor has a positive impact on hybrid nanofluid viscosity value, according to Equation (9). Because the factor coefficient is bigger than T, both solutions reveal that the factor is more successful against nanofluids, but viscosity temperature is more effective for the type of nanofluid utilized in viscosity temperature.

### 3.4. Contour and Surface Plots for Thermal Conductivity and Viscosity

**Figure 1:**
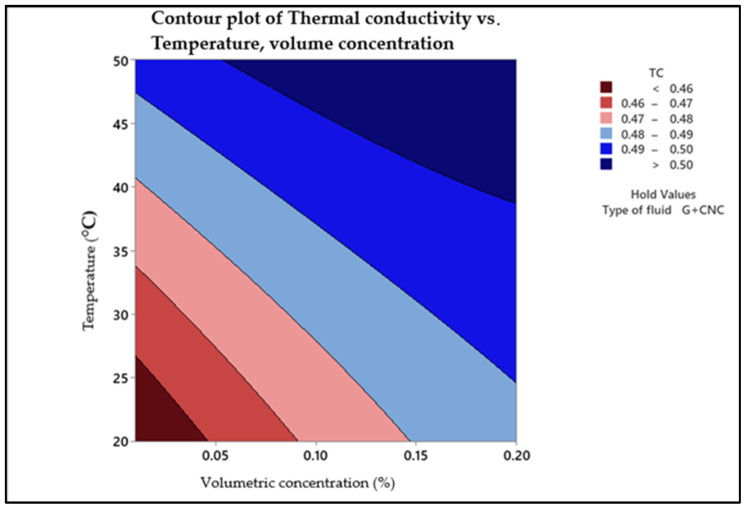
Contour plot of thermal conductivity.

In three-dimensional graphs and contours, Figure 1 depict temperature and volume concentration (%) influence on the thermal conductivity of a nanofluid. Curves and contours can be expected because of the strong influence of both sequential concentrations along with linear model.

The contour plots confirm that as temperature along with nanofluid concentration are both high, maximum thermal conductivity is obtained. This section can be found in the plot’s upper right corner. The interaction impact of temperature (*y*-axis) and nanofluid volume concentration (*x*-axis) on thermal conductivity is shown in Figure 1. It is observed that as the temperature and volume concentration rise, the thermal conductivity value increases. The falling tendency of thermal conductivity with nanoparticle concentration is extra visible during lower levels of nanofluid concentration, with a thermal conductivity value of 0.46 (W/m-k) at lower temperatures of 20–25 °C, as shown in the picture. The highest thermal conductivity of >0.50 (W/m-K) is at 0.20% volume concentration and temperature of 50 °C. The hold value for the type of fluid is hybrid nanofluid with nanoparticles of graphene nanoplatelets and cellulose nanocrystal. The random motions of nanoparticles increase as the temperature rises, causing energy to be transmitted quicker inside the nanofluid.

**Figure 2:**
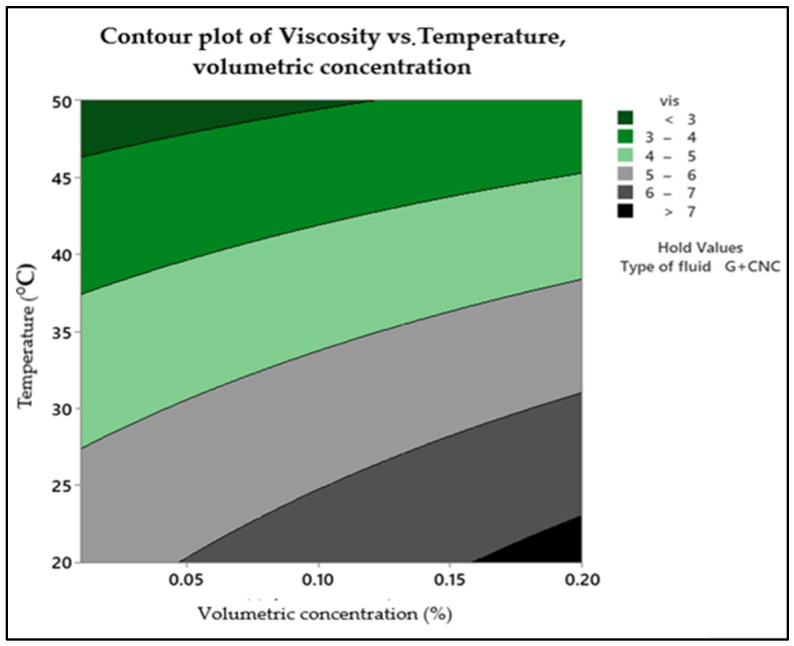
Contour plot of viscosity.

Viscosity statistical conditions for concentration and temperature are revealed in the contour plot. The contour plots are shown in Figure 2. The highest viscosity is reached when the temperature is low and the nanofluid concentration is high. This part is located in the lower right corner of the plot. Temperature (*y*-axis) along with nanofluid volume concentration (*x*-axis) interact to affect viscosity. It is understood from the plot that at highest temperature of 50 °C and lowest concentration of nanofluid, there is a minimum viscosity value ranging <3 (cP) as a hold value of type of fluid as is hybrid nanofluid with nanoparticles of graphene nanoplatelets and cellulose nanocrystal. Internal friction of a flowing fluid as a measure of fluid resistance to flow indicates higher temperatures and lower solid particles concentration/weight as indicated in the plot upper left corner. A fluid with a high viscosity resists motion because of the internal friction caused by its molecular structure. Low viscosity fluids flow easily because there is no friction created by their molecular structure.

**Figure 3:**
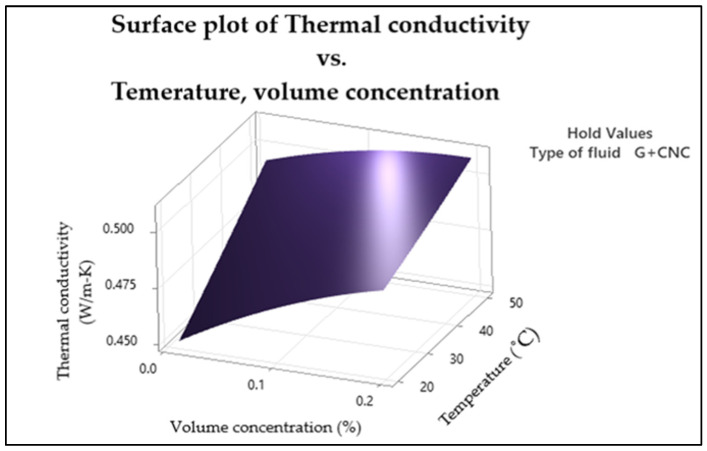
Thermal conductivity surface plot.

The response surface plot for thermal conductivity generated with the statistical data in the experimental part is shown in Figure 3. The quadratic effect that the volume concentration factor presented can be observed setting the temperature at its high level (50 °C). At this temperature, the peak thermal conductivity reached was 0.5 W/m-K at volume concentration of 0.2%. However, at 50 °C, and changing the volume concentration near to its lower level, it possibly reached a thermal conductivity in between 0.49 to 0.5 (W/m-K). This can be taken as the optimal point since it reduces the concentration from 0.2 to nearly 0.03%; while thermal conductivity is expected to be at its highest, it is desirable for viscosity to have a low value.

**Figure 4:**
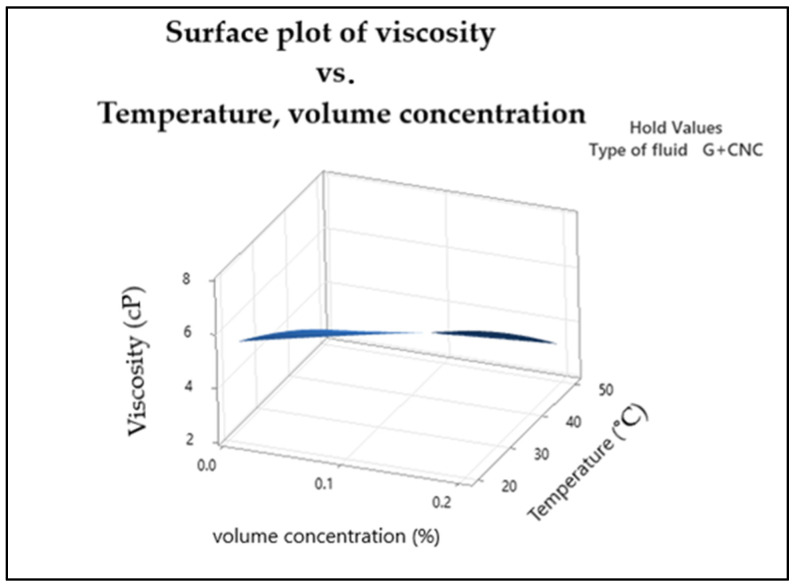
Surface plot of viscosity.

Figure 4 shows the response surface for representing the optimal conditions for fluid viscosity. The curvature of the graph increases as the temperature rises, reaching its highest point at a lower level of temperature and volume concentration. However, the curvature decreases as the temperature increases to its high level.

### 3.5. Pareto and Residual Plots for Thermal Conductivity and Viscosity

Pareto and residual plots are used in statistical analysis to identify the most significant factors contributing to a problem or issue. In the case of thermal conductivity and viscosity, Pareto and residual plots can be used to identify the most significant factors affecting these properties. A Pareto chart for thermal conductivity and viscosity would display the bars in descending order of frequency or magnitude, with the cumulative percentage of the total represented by a line graph. The bars would be arranged with the most significant factor and the least significant. The cumulative percentage line is the plot on the secondary axis. A residual plot for thermal conductivity and viscosity would display the residuals (the difference between the observed values and the predicted values) on the *y*-axis and the predicted values on the *x*-axis. The plot shows whether the residuals are randomly distributed around zero or whether there is a pattern to the residuals. A random distribution of residuals indicates that the model is a good fit for the data, while a pattern to the residuals indicates that there may be a problem with the model. Overall, Pareto and residual plots are used here for identifying the most significant factors affecting thermal conductivity and viscosity, and for evaluating the accuracy of models used to predict these properties.

**Figure 5:**
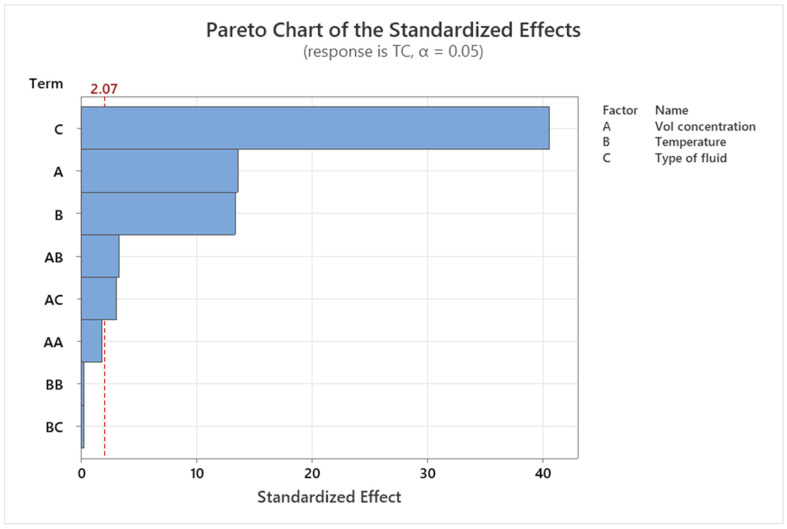
Pareto chart demonstration of the standardized effect for thermal conductivity of the nanofluid indicated with red dashed line.

A Pareto chart of the standardized effects was made to compare the importance of each effect. The responsive thermal conductivity of hybrid graphene nanoplatelets/cellulose nanocrystal/EG-water nanofluids is shown in Figure 5 as standardized effects plots. The range of data can be segmented into groups to create a Pareto chart. In order to evaluate the relative magnitude and statistical significance of both primary effects and their interactions, the Pareto chart also displays the relative importance of the effects. Everything that has an impact outside this reference line could be significant. Henceforth, the terms A, B, C, AB (Interaction of A and B), and AC (Interaction of A and C) are essential in the Pareto chart of the standardized effects, i.e., the factors nanofluid concentration (A), temperature (B), type of fluid (C), and interaction of AB and AC are significant, but the interactions of AA, BB, and BC are not significant for thermal conductivity. The most important factor is the categorical factor, which is the nanofluid type.

**Figure 6:**
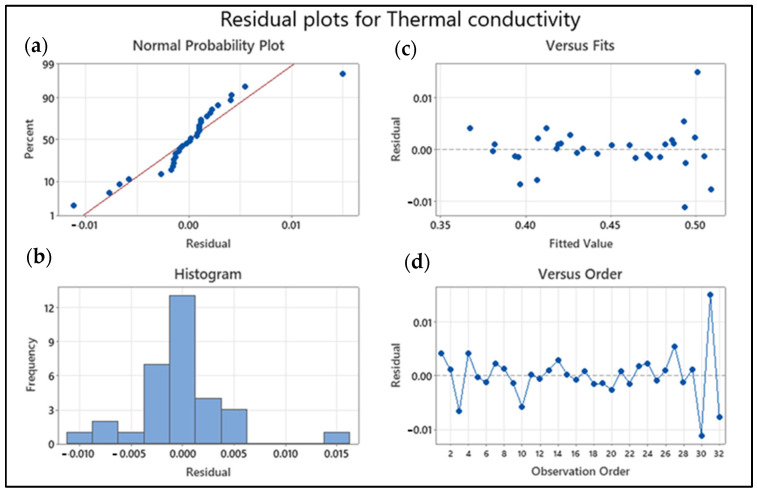
Thermal conductivity of nanofluids residual plots: (**a**) Normal probability plot with residuals (blue dots) vs. idealized normality (red line), (**b**) residual histogram, (**c**) residuals (blue dotted line) vs. fits (blue dotted path) plot, and (**d**) residuals (blue dotted line) vs. order of data (blue line) plot.

The minitab charts for thermal conductivity residual plots demonstrate the validity of the regression model’s assumptions with a mean error of zero. The normal probability plot (top left) verifies that error terms are normal as shown in Figure 6. The datasets are clustered around a straight line on the graph, showing that the error terms are relatively typical. As a result, the normality assumption is validated. The error terms are plotted against the fitted values in the Versus Fits graph (top right). At the top and bottom of the line, the data points appear to be evenly dispersed. The residual vs. fitted value plot and the residual vs. observation order plot both give information about how the response and error are related. The random behavior of the data points in these types of plots has been established as an indication of a good fit model, a fact that is commonly noticed in this circumstance.

**Figure 7:**
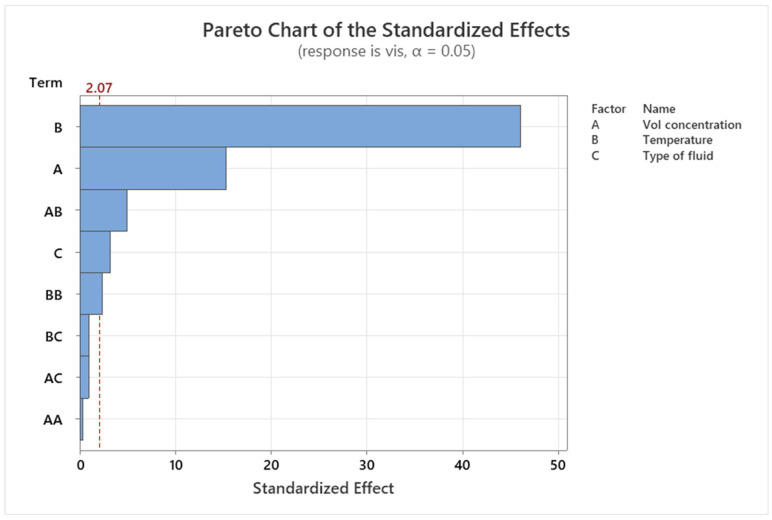
Pareto chart demonstration of the standardized effect for viscosity of the nanofluid indicated with red dashed line.

Figure 7 shows a Pareto chart of the standardized effects for viscosity. Standardized effects plots depict the responsive viscosity of hybrid graphene nanoplatelets/cellulose nanocrystal/EG-water nanofluids. The Pareto chart also depicts the relative importance of the effects, which may be used to determine the statistical significance of both the principal effects and their interactions, as well as their relative magnitude. Any influence that goes beyond this point of reference could be significant. Hence, in the Pareto chart of the standardized effects, the terms A, B, C, AB (Interaction of A and B), and BB (Interaction of B and B) are substantial, i.e., the attributed nanofluid concentration (A), temperature (B), type of fluid (C), and interaction of AB and BB are significant, but the interactions of AA, AC, and BC are not. The temperature identified in the Pareto chart of the standardized effects for viscosity is the most critical element. The linear and quadratic terms of temperature appear to have a substantial effect on viscosity, but their interaction effect is below the significance level and hence could be ignored when computing the projected viscosity response. The ANOVA analysis from Table 4 also demonstrates the statistical consequence of the system, with an F-value of 299.63 along with a *p*-value which is less than 0.0001.

**Figure 8:**
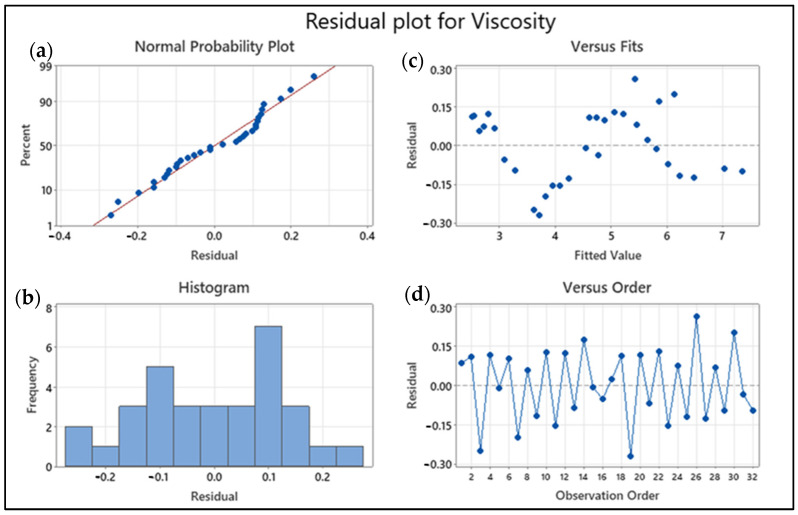
Viscosity of nanofluids residual plots: (**a**) Normal probability plot with residuals (blue dots) vs. idealized normality (red line), (**b**) residual histogram, (**c**) residuals (blue dotted line) vs. fits (blue dotted path) plot, and (**d**) residuals (blue dotted line) vs. order of data (blue line) plot.

Having a mean error value of 0, from the minitab graphs for viscosity, residual plots indicate the correctness of the regression model’s assumptions. The normal probability plot (top left) in Figure 8 indicates normal error terms. On the graph, the datasets are clustered around a straight line, indicating that the error terms are rather common. The normality of the plot depicted by bars and line with normal distribution is supported by the histogram plot for frequency and residual. As a result, the premise of normality is confirmed. In the versus fits graph, the error terms are displayed against the fitted values (top right). The data points appear to be uniformly distributed at the top and bottom of the line. Both the residual vs. fitted value plot and the residual vs. observation order plot provide insight into the relationship between response and error. The random behavior of data points in these types of plots has been established as an indicator of a good fit model, a fact that is widely observed in this situation.

### 3.6. Multi-Objective Optimization

**Figure 9:**
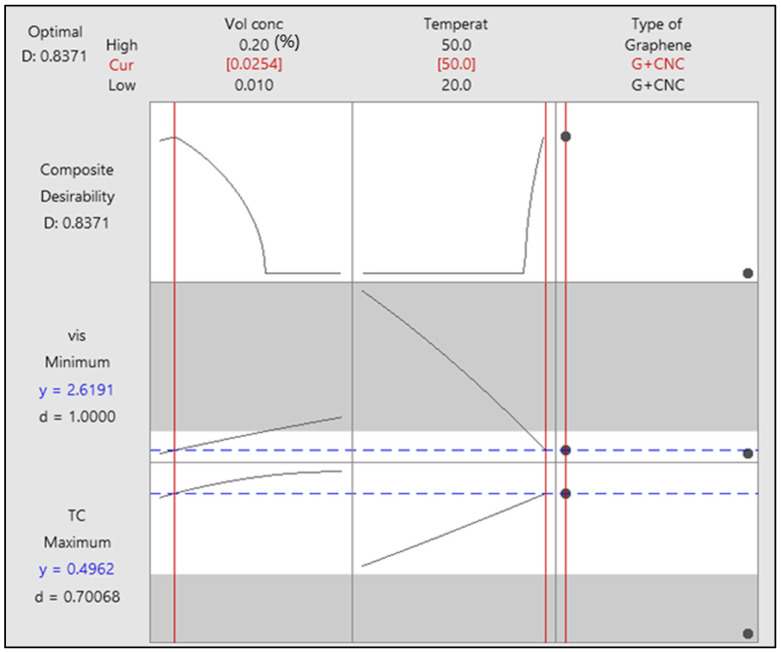
Optimization plot.

An optimization plot from response surface methodology (RSM) is a graphical representation of the response surface that shows the optimal values of the independent variables that maximize or minimize the response variable. The plot shows a contour plot of the response surface with the optimal values of the independent variables marked by a dot from Figure 9. The contour plot shows the relationship between the independent variables and the response variable, with the contours representing the levels of the response variable. The optimal values of the independent variables are determined by finding the maximum or minimum point on the contour plot.

The optimization plot is useful for identifying the optimal values of the independent variables that maximize or minimize the response variable, and for visualizing the relationship between the independent variables and the response variable. It can also be used to identify the regions of the response surface where the response variable is most sensitive to changes in the independent variables. The main advantage of adopting response surface methodology (RSM) is to alter the input constraints for improving response [40]. As stated earlier, the rising concentration increases the conductivity of the material, while rising temperature at a steady volume concentration increases thermal conductivity primarily before being nearly steady in the practices. Viscosity, similar to thermal conductivity, drops dramatically with temperature increase in a persistent volume concentration. This study’s aim is to identify the best combination of conductivity and viscosity.

The optimization graph of thermal conductivity and viscosity reactions are seen in Figure 9. The plot’s optimum values for thermal conductivity and viscosity are 0.4962 W/m-K and 2.6191 cP. The important parameters are 50 °C and 0.0254%, GNP/CNC is the most important categorical factor, and relevant parameters are concentration, temperature, and kind of nanofluid. The composite value is 0.8371 in this plot.

An experiment was performed utilizing the optimum factors to evaluate the thermal conductivity and viscosity to confirm the optimized solutions. Fluids thermal properties were assessed and contrasted to the model’s predicted findings under ideal conditions. The findings in Table 6 show that the standard values can accurately anticipate the best investigational circumstances.

### 3.7. Applications of Hybrid Nanofluids

Optimized nanofluids can be used in various heat transfer applications such as cooling systems in electronic devices, heat exchangers, solar collectors, and automotive engines. The improved thermal conductivity and viscosity of the hybrid nanofluids can enhance the heat transfer efficiency of these systems, leading to better performance and energy savings. Additionally, the use of nanofluids can also reduce the size and weight of heat transfer equipment, making it more compact and cost-effective.

## 4. Conclusions

According to the results from the thermophysical properties of hybrid nanofluids comprising of graphene nanoplatelets and graphene nanoplatelets/cellulose nanocrystal nanoparticles in the base fluid of ethylene glycol and water (60:40), the optimized parameter values are derived, and the main conclusions are as follows.

Based on the empirical technique used in this study, the actual and projected results of thermal conductivity and viscosity are highly correlated, with R^2^ values exceeding 80% for both responses. The optimization plot shows that the optimal values of thermal conductivity and viscosity are 0.4962 W/m-K and 2.6191 cP, respectively, with the important parameters being 50 °C and 0.0254%, and the categorical factor being GNP/CNC. The appropriate parameters for achieving these optimal values are volume concentration, temperature, and the type of nanofluid used. The composite obtained from the graph is 0.8371. The experimental validation results indicate that the system can accurately predict the best experimental circumstances for achieving the desired thermal conductivity and viscosity values. Overall, these findings suggest that the empirical technique used in this study is a reliable method for predicting and optimizing thermal conductivity and viscosity in nanofluids.

## Figures and Tables

**Table 1:** Various levels of factors.

Type of Factors	Factors	−1	+1
Continuous factors	Temperature (°C)	20	50
	Volume Concentration (%)	0.01	0.2
Categorical factors	Type of Nanolubricant	GNP	GNP/CNC

**Table 2:** Design of the experiments and its findings.

Std Order	Temperature °C (T)	Volume Concentration % (Ø)	Type of Nanofluid	Thermal Conductivity (k) W/m-K	Viscosity (cP)
1	20	0.01	Graphene	0.371	5.54
2	30	0.01	Graphene	0.382	4.72
3	40	0.01	Graphene	0.390	3.37
4	50	0.01	Graphene	0.416	2.62
5	20	0.05	Graphene	0.380	5.79
6	30	0.05	Graphene	0.392	4.98
7	40	0.05	Graphene	0.409	3.63
8	50	0.05	Graphene	0.422	2.70
9	20	0.10	Graphene	0.394	6.11
10	30	0.10	Graphene	0.400	5.34
11	40	0.10	Graphene	0.418	3.92
12	50	0.10	Graphene	0.429	2.93
13	20	0.20	Graphene	0.420	6.94
14	30	0.20	Graphene	0.428	6.02
15	40	0.20	Graphene	0.434	4.53
16	50	0.20	Graphene	0.441	3.04
17	20	0.01	G+CNC	0.451	5.67
18	30	0.01	G+CNC	0.462	4.85
19	40	0.01	G+CNC	0.477	3.44
20	50	0.01	G+CNC	0.491	2.66
21	20	0.05	G+CNC	0.461	5.95
22	30	0.05	G+CNC	0.471	5.18
23	40	0.05	G+CNC	0.487	3.79
24	50	0.05	G+CNC	0.501	2.79
25	20	0.10	G+CNC	0.470	6.35
26	30	0.10	G+CNC	0.483	5.68
27	40	0.10	G+CNC	0.498	4.11
28	50	0.10	G+CNC	0.503	2.98
29	20	0.20	G+CNC	0.488	7.26
30	30	0.20	G+CNC	0.482	6.33
31	40	0.20	G+CNC	0.515	4.73
32	50	0.20	G+CNC	0.501	3.18

**Table 3:** ANOVA (analysis of variance) result for thermal conductivity.

Source	DF	Seq SS	Adj SS	Adj MS	F-Value	*p*-Value
Model	8	0.057066	0.057066	0.007133	273.23	0.000
Linear	3	0.056434	0.052458	0.017486	669.78	0.000
Vol concentration	1	0.004741	0.004831	0.004831	185.03	0.000
Temperature	1	0.005377	0.004658	0.004658	178.42	0.000
Type of fluid	1	0.046315	0.042970	0.042970	1645.91	0.000
Square	2	0.000092	0.000092	0.000046	1.75	0.196
Vol concentrationVol concentration	1	0.000089	0.000089	0.000089	3.42	0.077
Temperature*Temperature	1	0.000002	0.000002	0.000002	0.09	0.773
2-Way Interaction	3	0.000541	0.000541	0.000180	6.91	0.002
Vol concentration*Temperature	1	0.000287	0.000287	0.000287	11.00	0.003
Vol concentration*Type of fluid	1	0.000252	0.000252	0.000252	9.65	0.005
Temperature*Type of fluid	1	0.000002	0.000002	0.000002	0.07	0.794
Error	23	0.000600	0.000600	0.000026		
Total	31	0.057667				

**Table 4:** Analysis of variance result for viscosity.

Source	DF	Seq SS	Adj SS	Adj MS	F-Value	*p*-Value
Model	8	59.4256	59.4256	7.4282	299.63	0.000
Linear	3	58.6352	58.6423	19.5474	788.49	0.000
Vol concentration	1	5.8737	5.8025	5.8025	234.06	0.000
Temperature	1	52.5252	52.5840	52.5840	2121.09	0.000
Type of fluid	1	0.2363	0.2559	0.2559	10.32	0.004
Square	2	0.1440	0.1440	0.0720	2.90	0.075
Vol concentration*Vol concentration	1	0.0031	0.0031	0.0031	0.13	0.725
Temperature*Temperature	1	0.1409	0.1409	0.1409	5.68	0.026
2-Way Interaction	3	0.6464	0.6464	0.2155	8.69	0.000
Vol concentration*Temperature	1	0.6025	0.6025	0.6025	24.30	0.000
Vol concentration*Type of fluid	1	0.0214	0.0214	0.0214	0.86	0.363
Temperature*Type of fluid	1	0.0226	0.0226	0.0226	0.91	0.350
Error	23	0.5702	0.5702	0.0248		
Total	31	59.9958				

**Table 5:** Summary of the thermal conductivity and viscosity model.

Model	S	R-Square	R-Square (Adjacent)	PRESS	R-Square (Prediction)	AICc	BIC
Thermal conductivity	0.0051095	98.96%	98.60%	0.0012977	97.75%	−226.99	−222.80
Viscosity	0.157452	99.05%	98.72%	1.04063	98.27%	−7.59	−3.41

**Table 6:** Optimal condition values.

Optimum Results	Temperature (°C)	Concentration (%)	Type of Nanofluid	Experimental Value	Predicted Value	ARE%
Thermal conductivity (W/m-K)	50	0.0254	GNP/CNC	0.495443	0.4962	0.16148
Viscosity (cP)	50	0.0254	GNP/CNC	2.7134	2.6191	3.4753

## Data Availability

Not applicable.
